# Neoadjuvant Treatments for Pancreatic Ductal Adenocarcinoma: Where We Are and Where We Are Going

**DOI:** 10.3390/jcm12113677

**Published:** 2023-05-25

**Authors:** Alessandro Coppola, Tommaso Farolfi, Vincenzo La Vaccara, Immacolata Iannone, Francesco Giovinazzo, Elena Panettieri, Mariarita Tarallo, Roberto Cammarata, Roberto Coppola, Damiano Caputo

**Affiliations:** 1 Department of Surgey, Sapienza University of Rome, Viale Regina Elena 291, 00161 Rome, Italy; 2 General Surgery, Fondazione Policlinico Universitario Campus Bio-Medico, 00128 Rome, Italy; 3 General Surgery, Università Campus Bio-Medico di Roma, 00128 Rome, Italy; 4 General Surgery and Liver Transplant Unit, Fondazione Policlinico Universitario A. Gemelli IRCCS, Largo A. Gemelli 8, 00168 Rome, Italy; 5 Hepatobiliary Surgery Unit, Fondazione Policlinico A. Gemelli IRCCS, Università del Sacro Cuore, 00168 Rome, Italy

**Keywords:** pancreatic ductal adenocarcinoma, neoadjuvant treatment, CA 19-9, upfront surgery, resectable pancreatic ductal adenocarcinoma

## Abstract

Background: Pancreatic ductal adenocarcinoma (PDAC) represents a challenging disease for the surgeon, oncologist, and radiation oncologist in both diagnostic and therapeutic settings. Surgery is currently the gold standard treatment, but the role of neoadjuvant treatment (NAD) is constantly evolving and gaining importance in resectable PDACs. The aim of this narrative review is to report the state of the art and future perspectives of neoadjuvant therapy in patients with PDAC. Methods: A PubMed database search of articles published up to September 2022 was carried out. Results: Many studies showed that FOLFIRINOX or Gemcitabine-nab-paclitaxel in a neoadjuvant setting had a relevant impact on overall survival (OS) for patients with locally advanced and borderline resectable PDAC without increasing post-operative complications. To date, there have not been many published multicentre randomised trials comparing upfront surgery with NAD in resectable PDAC patients, but the results obtained are promising. NAD in resectable PDAC showed long-term effective benefits in terms of median OS (5-year OS rate 20.5% in NAD group vs. 6.5% in upfront surgery). NAD could play a role in the treatment of micro-metastatic disease and lymph nodal involvement. In this scenario, given the low sensitivity and specificity for lymph-node metastases of radiological investigations, CA 19-9 could be an additional tool in the decision-making process. Conclusions: The future challenge could be to identify only selected patients who will really benefit from upfront surgery despite a combination of NAD and surgery.

## 1. Introduction

Pancreatic ductal adenocarcinoma (PDAC) represents a challenging disease in either diagnosis or treatment [1]. It currently represents the fourth highest cause of cancer mortality and, due to the important therapeutic advances implemented for other cancers, it is expected to become the second highest cause in the coming years [2]. Surgery is currently the gold standard treatment for patients with PDAC [3]. Unfortunately, only a small percentage of patients (below 20%) could be candidates for surgical treatment at the time of diagnosis [1].

Upfront surgery followed by adjuvant chemotherapy represents the curative treatment in PDAC patients with radiologically resectable cancer [4].

Unfortunately, despite this being the gold standard treatment, 5-year overall survival remains low, with rates of approximately 20% [5].

On the contrary, improving results in terms of disease-free survival (DFS) and median overall survival were observed in the treatment of borderline resectable (BR) and locally advanced (LA) PDAC by the adoption of preoperative neoadjuvant treatments (NAD).

NAD followed by surgery represents the standard of care in borderline resectable and locally advanced PDAC patients. The purposes of NAD are downsizing and/or vascular involvement, treating occult lymph nodal and distant micro-metastases and assessing the biological chemosensitivity of the tumour [6].

Hence, based on the results obtained in BR- and LA-PDAC, NAD is gaining momentum, even in patients with resectable PDAC instead of upfront surgery [7]. Furthermore, in recent years, the concept of resectability has been changing. The International Study Group of Pancreatic Surgery (ISGPS) and the International Association of Pancreatology (IAP) introduced the concept of biological resectability [8]. Therefore, the anatomical criteria may no longer be sufficient for the correct identification of patients who are eligible for neoadjuvant treatment. The aim of this narrative review is to report the state of the art and future prospects of neoadjuvant therapy in patients with PDAC.

## 2. Materials and Methods

A PubMed database search of articles published up to September 2022 was carried out. Different combinations of the following terms were used: neoadjuvant treatments and pancreatic cancer, pancreatic adenocarcinoma, FOLFIRINOX, CA 19-9, nodal involvement and overall survival. Only articles published in English with available full text were considered without limitation concerning article types (original articles, review, etc.). References reported in the selected articles were also considered as other bibliographic sources.

## 3. Types of Neoadjuvant Treatments

### 3.1. FOLFIRINOX

The FOLFIRINOX regimen, composed of folinic acid (leucovorin), a vitamin B derivative used to raise the effects of 5-fluorouracil (5-FU), 5-FU, a pyrimidine analogue, Irinotecan (Camptosar), a topoisomerase inhibitor and oxaliplatin (Eloxatin) has been largely used in metastatic disease. Based on those results, this regimen has been proposed in a neoadjuvant setting too.

Starting from 2011, the efficacy of this treatment was validated by Conroy et al. in a study involving more than 500 PDAC patients; they showed a median survival of 11.1 months in the FOLFIRINOX group compared to 6.8 months in the gemcitabine alone group [9].

Unfortunately, this type of therapy is affected by high rates of toxicity; therefore, a modified therapy regimen has been proposed. “Modified FOLFIRINOX” (mFOLFIRINOX), without bolus fluorouracil, has shown comparable results in neoadjuvant settings despite it not being directly compared with FOLFIRINOX [10].

The multicentre PRODIGE trial, published in 2018, confirmed the efficacy of mFOLFIRINOX versus gemcitabine alone. In the trial, 493 patients randomly assigned to mFOLFIRINOX or gemcitabine demonstrated an improvement in the 3-year disease-free survival (DFS) rate (39.7% in the modified FOLFIRINOX group vs. 21.4% in the gemcitabine group). Additionally, the median DFS was significantly different (21.6 months vs. 12.8 months) [11].

Suker et al., in a review of 11 studies and over 300 locally advanced PDAC, showed that neoadjuvant FOLFIRINOX for LA-PDAC allowed more than 25% of patients to undergo surgical resection with a negative margin (R0) resection rate of 74% [12].

### 3.2. Gemcitabine/Nab-Paclitaxel

Based on preclinical studies conducted in murine models, albumin-bound paclitaxel particles (nab-paclitaxel [Abraxane]) in combination with gemcitabine had anti-tumour activity increasing the intratumoral concentration of gemcitabine [13]. In 2011, Von Hoff et al. conducted a phase 1–2 clinical trial in untreated metastatic PDAC, showing that gemcitabine-abraxane had promising results in terms of OS with acceptable levels of adverse events [14]. 

A few years later, a phase 3 clinical trial was conducted by the same authors. In a group of 861 metastatic PDAC patients, the OS was 8.5 months in the nab-paclitaxel group compared to 6.7 months in the gemcitabine group. Additionally, the median progression-free survival was higher in the nab-paclitaxel group (5.5 months vs. 3.7 months). The most common adverse events were neutropenia and neuropathy [15].

To date, no multicentre studies have been reported in the neoadjuvant setting. In 2016, Ielpo et al. analysed a group of 11 patients with borderline resectable PDAC treated with the combination of gemcitabine/nab-paclitaxel, showing a resection rate of 73% with an R0 of 100% after surgery [16].

### 3.3. Gemcitabine/Docetaxel/Capecitabine (GTX)

The three-drug regimen composed of gemcitabine/docetaxel/capecitabine (GTX) was described for the first time in 2004 at Columbia University [17].

As for the gemcitabine/nab-paclitaxel regimen, no multicentre studies have been conducted to date. In 2018, Sherman described 34 patients with BR- or LA-PDAC treated with GTX and showed that 100% of those with venous involvement and 85% with arterial involvement underwent radical resection [18].

Analysing the economic aspect, this regimen has significantly reduced costs when compared with the other neoadjuvant drugs; however, to date, it is still far from being widely adopted [10].

### 3.4. Radiation Therapy

The role of radiation therapy is still debated; however, in the last decades, important advances have been made in this field.

Regarding LA-PDAC, following that which was previously established by the LAP07 trial in 2016 [19], the current recommended treatment is a standard dose of chemoradiation to 54 Gy in 30 fractions or stereotactic body radiation therapy (SBRT) to 25–33 Gy in 5 fractions. In the LAP07 trial, this regimen was established comparing an induction chemotherapy followed by chemoradiation (with capecitabine) with an induction chemotherapy alone. The chemoradiation group showed a local control benefit, but no changes in overall survival (OS) were observed.

Promising perspectives have been showed in several prospective phase 1/2 studies on the radio-sensitisation using molecularly targeted agents to improve the efficiency of radiation therapy [20].

For BR-PDAC, there are no recommended radiation therapy regimens to date. Several trials are ongoing to investigate the optimal neoadjuvant regimen, which will probably be identified in the next several years.

## 4. Results of NAD in Locally-Advanced-PDAC

LA-PDAC has historically been considered ineligible for surgical resection due to both anatomical characteristics and the biological aggressiveness of the tumour.

In 2013, a Japanese trial evaluated the efficiency of neoadjuvant treatments in LA-PDAC. In their study, using a combination of tegafur, gimeracil and oteracil potassium in a neoadjuvant setting, Ikeda et al. showed a 27% rate of partial response, but a downstaging of the tumour led to surgical resection in only 3% of cases [21].

Blazer et al. published a retrospective study on 43 patients with LA-PDAC treated with neoadjuvant FOLFIRINOX. In their work, after therapy, 25 patients (44%) were eligible for surgical resection and postoperative results showed an R0 of 91% after surgery. No significant event of toxicity was reported [22].

These results were confirmed by Nanda et al. in a single-centre retrospective study. In a series of 29 patients with LA-PDAC treated with FOLFIRINOX, 41.3% of patients underwent surgery with an R0 of 83% [23].

In the same year, Marthey et al. published a multicentre phase 2 trial that included 77 LA-PDAC patients treated with FOLFIRINOX with or without neoadjuvant radiation therapy. Treatment response was evaluated based on the RECIST (Response Evaluation Criteria in Solid Tumours) criteria. Resection rates after therapy were 36% with an R0 of 86%. They observed a median overall survival of 22 months [24].

In 2016, Suker et al. published a systematic review that may represent, to date, the best available evidence of FOLFIRINOX in LA-PDAC patients. They confirmed the previously reported results: Of the 315 patients included in the review, 25.9% had surgical resection with an R0 resection reported in 60 (74%) patients. The median overall survival was 24.2 months [12]. In 2019, Gemenetzis et al. confirmed that, in an extremely selective group of LA-PDAC patients (around 20%), surgical resection after neoadjuvant therapy is feasible and associated with significantly longer median overall survival (Table 1) [25]. 

## 5. Results of NAD in Borderline Resectable PDAC

Therapeutic strategies for BR-PDAC have represented a challenge for pancreatic surgeons.

Since 2010, the role of neoadjuvant treatment in BR-PDAC has been investigated. Laundry et al. performed one of the first published trials, comparing two different regimens of neoadjuvant radio-chemotherapies. In their cohort of 21 BR-PDAC, gemcitabine 500 mg/m^2^ weekly for 6 weeks with concomitant RT led to a resection rate of 30% with an overall survival of 19.4 months; this was compared to a resection rate of 18% with an OS of 13.4 months when chemotherapy was given alone and followed by RT [26].

In 2013, Kim et al. showed interesting results in a larger cohort including 68 BR-PDAC patients treated with gemcitabine + oxaliplatin with 30 gray radiotherapy. In their work, 43 patients underwent surgery (resection rate of 63%) with an R0 resection rate of 84%. An OS of 18.2 months was observed [27].

In 2016, Katz published the results of the Alliance for Clinical Trials in Oncology Trial A021101, a prospective multicentre single-arm trial, in which 22 BR-PDAC patients were treated with modified FOLFIRINOX. Here, four cycles of mFOLFIRINOX followed by 5.5 weeks of radiotherapy with concomitant capecitabine were given. In this trial, 15 of the 22 (68%) patients underwent surgery with an R0 resection in 14 patients (93%). An OS of 21.7 months was found, which represents the highest OS in BR-PDAC [28].

In 2018, two other trials were presented. The first was published by Murphy et al. and included only BR-PDAC treated with 8 cycles of FOLFIRINOX, confirming the previously cited results (66% resection rate with an R0 resection of 97%) [29].

The second one, PREOPANC, is a randomised phase 3 trial which aimed to compare upfront surgery with neoadjuvant treatment followed by surgical resection in resectable and BR-PDAC [7].

Their preliminary results after two years of follow-up showed a comparable resectability rate between the two groups (61% in the upfront surgery group and 72% in the neoadjuvant group (*p* = 0.058) but with a doubled R0 resection rate (71% in patients who received preoperative chemoradiotherapy and 40% in patients who underwent upfront surgery). In more detail, promising results were obtained in the BR-PDAC subgroup. OS in the neoadjuvant group was 17.6 months compared to 13.2 months in the upfront surgery group, with a comparable disease-free survival.

Their study showed a higher disease-free survival as well as lower rates of pathological lymph nodes, perineural invasion and venous invasion in the BR-PDAC cases treated with preoperative chemotherapy.

Furthermore, survival analysis of patients who received neoadjuvant treatment showed a higher OS survival (35.2 vs. 19.8 months; *p* = 0.029).

Those results were later confirmed in the long-term results published in 2022, both in resectable and borderline resectable PDAC.

Even if no significant difference was found in median survival (15.7 months vs. 14.3 months), the 5-year OS rate at the 5-year follow-up was 20.5% in the neoadjuvant chemoradiotherapy group and 6.5% in the upfront surgery group [30].

The last published NCCN guidelines recommend neoadjuvant treatment as the first-line treatment for BR-PDAC, even if resection is technically feasible.

Other prospective trials are ongoing, like PRODIGE 44, which is expected to finish in 2026 and is studying the role of mFOLFIRINOX followed by capecitabine radio-chemotherapy (Table 2).

## 6. Results of NAD in Resectable PDAC

Upfront surgery followed by adjuvant chemotherapy still represents the gold standard treatment for resectable PDAC. The NCCN guidelines recommend administering a limited NAD scheme in highly selected subgroups of patients [31].

Nonetheless, in recent years, driven by the excellent results obtained from neoadjuvant treatments in the other PDAC categories, increasing interest has been placed on the use of neoadjuvant treatments in resectable PDAC.

To date, there have not been many published multicentre randomised controlled trials comparing upfront surgery with NAD in resectable PDAC. The first is the previously mentioned 2020 PREOPANC. At the two-year control, in the subgroup of resectable PDAC, no differences in OS, DFS or resection rate were found. Median OS was 14.6 months in the NAD cohort versus 15.6 in the upfront surgery. Resection rate (68% vs. 79%) and DFS (9.2 vs. 9.3 months) were also comparable in the two populations [7].

However, the long-term results have changed the perspectives of this multicentre trial, highlighting that the OS was higher in the NAD group (both resectable and borderline resectable) compared to the upfront surgery group (20.5% vs. 6.5%) at the five-year follow-up [30].

The second is PREP-02, a multicentre randomised trial published by Motoi et al. [32]. The main aim of this trial was to confirm the superiority of gemcitabine and S-1 neoadjuvant chemotherapy compared to upfront surgery for resectable PDAC. A higher OS was found after gemcitabine-based neoadjuvant chemotherapy. No differences in terms of resection rate, margin status and morbidity were found [32].

In 2008, in a non-randomised study including 28 patients with resectable PDAC treated with gemcitabine plus cisplatin, Heinrich et al. showed a resection rate of 89% with an R0 of 80% after pancreatoduodenectomy and a median OS of 26.5 months; this was much higher than for other OS published during the same period [33].

Those data were confirmed in 2014 by O’Reilly et al. in a single-arm phase 2 trial with a cohort of 38 resectable PDAC patients treated with gemcitabine plus oxaliplatin. In their study, the OS was 27.2 months [34].

The role of neoadjuvant radiotherapy in addition to chemotherapy in resectable PDAC remains unclear. In 2015, two studies were published showing comparable rates. In particular, they found that a NAD treatment composed of radio-chemotherapy does not reduce resection rates but instead increases the median OS [35,36].

No randomised trials with FOLFIRINOX have been published to date. The PANACHE01 multicentre trial is ongoing, which is exploring the results of this regimen in resectable PDAC [37] (Table 3).

## 7. Discussion

In 2014, the International Study Group of Pancreatic Surgery defined radiological classification criteria for pancreatic tumours as resectable, borderline resectable, locally advanced and metastatic [38].

This classification, based on the relation between the tumour and the principal vascular structure around the pancreas, superior mesenteric vein and artery allows a more precise and personalised therapeutic strategy for each patient with PDAC.

Nowadays, with these three classes, patients were selected for two main therapeutic strategies: surgery upfront and neoadjuvant treatments followed by surgery.

Neoadjuvant therapy has placed itself in this panorama, driven by the excellent results obtained with other types of tumours (e.g., oesophagus, rectum and stomach).

NAD has been demonstrated to be safe for borderline resectable pancreatic cancer without increasing short-term post-operative complications [39].

As reported by Okabayashi et al., NAD has the potential to increase the R0 rate after surgery and constitute an early treatment of micro-metastases. Furthermore, the treatment window makes it possible to identify patients affected by very aggressive forms of tumours who would not benefit from such invasive surgical treatment [40].

In addition, NAD could allow patients to improve their performance status by avoiding operations during states of nutritional deficiency or sarcopenic status, which are well-known preoperative risk factors for postoperative complications and survival outcomes [41].

Another important role that NAD could play in resectable PDAC is the treatment of micro-metastatic disease and lymph nodal involvement (N+).

Recently, Heidelberg’s group demonstrated that lymph node involvement is the main factor impacting the survival of patients with upfront resected PDAC [42].

As reported by Ye et al. in a metanalysis published in 2020 of 11 studies, including more than 8000 PDAC patients, NAD reduced the number of N+ at the pathological examination, both for patients treated with gemcitabine-based therapy and for those undergoing 5-FU-based treatment [43].

To date, NAD has gained an important role in borderline resectable and locally advanced pancreatic cancers; in the former, with the aim of increasing the percentages of R0, while in the latter, to obtain a downstaging of the disease [44].

The Dutch Pancreatic Cancer Group demonstrated how neoadjuvant therapy had a great impact, especially on the BR-PDAC group. The median OS of patients with BR-PDAC is comparable with that of patients with resectable PDAC treated with upfront surgery [30].

The interesting aspect underlined by the PREOPANC trial is that the efficacy of NAD is time related. In the short-term, the survival outcomes between the NAD group and upfront surgery are comparable. However, the 5-year survival analysis showed an effective benefit from NAD in terms of OS (5-year OS rate 20.5% in the NAD group vs. 6.5% in the upfront surgery one).

In 2019, the results of a Japanese trial were published, demonstrating interesting results for NAD in resectable PDAC. After gemcitabine-based NAD, patients showed a high overall survival rate compared with upfront surgery (2-year OS rate of 55.9% in the control group and 74.6% in the neoadjuvant group); no difference was found in terms of resection rate, margin status or morbidity [32]. However, all previously reported findings are not sufficient to prompt anatomically resectable PDAC patients to undergo NAD.

Detractors of neoadjuvant treatments in resectable upfront PDAC have concerns regarding the risk of some patients failing to undergo surgery due to the toxicity of therapy. No data are currently available to clearly estimate this risk.

On the other hand, it has been reported that patients who underwent upfront resection have a risk of not being fit for systemic therapy after surgery due to the non-negligible rates of postoperative complications. In these cases, the adjuvant therapy could not start two months after surgery and, in some cases, patients were unable to start any kind of therapy at all [45].

Performing preoperative treatments could allow almost all patients to complete systemic therapy by also selecting the biological aggressiveness and chemosensitivity of the tumour, thus avoiding unnecessary surgery in some patients.

In order to select patients for NAD, avoiding unnecessary therapy in very early PDAC stages, the concept of biological resectability is gaining interest. With this concept, other factors in addition to the anatomical aspect were taken into consideration. For these reasons, the 2017 IAP consensus proposed considering not only patients with anatomical characteristics but also other classes of patients as being BR resectable. The criteria adopted alone or in combination were the following: anatomical, biological and conditional. In the biological criteria, preoperative suspicious findings of lymph nodal involvement, unproven distant metastases or elevated values of serum carbohydrate antigen (Ca) 19-9 should be considered as characteristics that move preoperative staging from upfront resectable to BR resectable PDAC [46].

Additionally, NCCN guidelines from 2022 consider NAD in anatomical resectable PDAC in the presence of high-risk features, including elevated CA 19-9, large tumours, regional lymphadenopathies, excessive weight loss and extreme pain [31].

In this scenario, CA 19-9, the only marker approved for clinical use by the Food and Drug Administration (FDA) for PDAC, could represent an additional tool in the decision-making process of resectable PDAC treatments.

Until a few years ago, CA 19-9 was considered a useful marker in the evaluation of adjuvant therapy after surgery or for the evaluation of the efficacy of systemic therapy in patients with metastatic disease [47].

However, several reports in the literature are also underlining the role of CA 19-9, alone or in combination with other biological markers, in the preoperative staging and prognosis prediction of patients with resectable PDAC [48].

High preoperative serum levels of CA 19-9 have been reported to be a reliable tool in predicting prognosis in PDAC patients from 2006 when Ferrone et al. showed that preoperative CA 19-9 > 1000 U/mL had a worse OS compared to those with CA 19-9 levels lower than 1000 U/mL (12 months vs. 28 months) [49].

In 2019, those results were confirmed and implemented by Mattiucci et al. They divided the population study into four groups based on preoperative CA 19-9 serum levels and found that the groups with the worse OS and disease-free survival were those with presurgical CA 19-9 between 100 and 353 and >353 U/mL [50].

Moreover, in recent years, numerous studies have been published exploring the prognostic role of preoperative serum levels of CA 19-9. In 2020, Fiore et al. studied a series of 120 patients and reported a 6-times higher risk of early recurrence in patients with a preoperative serum level of CA 19-9 > 698 U/mL [47].

In 2021, Coppola et al. published a retrospective study involving 165 PDAC patients who underwent surgery, showing how preoperative serum levels of CA 19-9 ≥ 32 U/mL were significantly related to nodal involvement at the final pathological report. This relationship was only observed in the presence of normal serum albumin levels.

Moreover, CA 19-9 > 730 U/mL was significantly found in PDAC patients with positive resection margins [51].

In 2021, Hua et al. showed a normogram based on both serum markers (not only CA 19-9 but also CA 125, CA 50 and CA 242) and radiological findings. The resulting nomogram could predict lymph node involvement [52]. CA 19-9 can also be used to evaluate the response to NAD, as shown by Takahashi et al. [53].

In this retrospective series of 407 patients, the normalisation of serum levels of CA 19-9 after NAD represents a significant positive prognostic factor.

However, despite its utility, CA 19-9 was not sufficient to identify candidate patients with anatomically resectable PDAC for NAD. For this reason, other markers are under evaluation alone or in combination with CA 19-9.

Moreover, it is well-known that there is a non-negligible number of patients who are CA 19-9 non-secretors. As a result, markers like DUPAN-2 could be adopted in this class of patients.

In a study published by Omiya et al., with a series of more than 900 PDAC patients with LA-PDAC, DUPAN-2 serum levels >2000 U/mL after NAD were shown to be an unfavourable predictive factor comparable to CA 19-9 serum levels >500 U/mL in CA 19-9 secretors [54].

Based on the actual scientific evidence, NAD should be the first-line therapy for borderline and locally advanced PDAC. Biologically borderline PDAC should also be considered a candidate for NAD treatment. However, the staging methods and technologies are not currently completely able to clearly determine the biological status. Further studies are required to clarify the unsolved staging issue for the better selection of patients.

Moreover, the different trials identified in the literature were performed with several different chemotherapy regimens and with or without associated radiotherapy, thus preventing any definitive conclusions from being drawn. As we can assume that we do not have enough data to consider NAD as the first-line treatment for all PDAC, we can also suppose that NAD does not impact on the chance to achieve curative surgery in all the reported studies.

## 8. Conclusions

Pancreatic cancer remains one of the diseases with the highest mortality rate. The results presented in this narrative review demonstrate how the adoption of neoadjuvant therapies is proving to be a fundamental element in the treatment of PDAC. These advantages are well reported in borderline and locally advanced tumours. Still debatable are the results in the early stages. Consequently, improvements in preoperative staging are required in order to discriminate really early stage PDAC from anatomically early stage PDAC biologically advanced disease, e.g., with lymph node metastasis. Neoplastic markers could drive physicians in this selection but, so far, no definitive conclusions can be drawn. The development of new chemotherapy drugs and radiotherapy schemes are the core of this success. Neoadjuvant chemotherapy alone seems to report the same or better outcomes in terms of 90-day mortality and overall survival. On the other hand, in other studies, patients treated with neoadjuvant chemoradiotherapy achieved higher R0 rates.

In addition, neoadjuvant therapy currently improves the survival of patients with PDAC by allowing the surgical selection of those patients with less biologically aggressive tumours, both locally and in distant tumour spread.

Neoadjuvant treatments are definitely candidates which may play an important role in the fight against this fatal and challenging disease.

## Figures and Tables

**Table 1:** Main characteristics and findings of studies reporting data about NAD in LA-PDAC.

Ref. n°	Author	Year	N° of Patients	Type	Concept Resumed
[21]	Ikeda, M.	2013	60	Multicenter phase II trial	27% rate of partial response (only a 3% of downstaging) using a combination of tegafur, gimeracil, and oteracil potassium in a neoadjuvant setting of the tumor led to surgical resection
[22]	Blazer, M.	2015	43	Retrospective study	After neoadjuvant FOLFIRINOX, 25 patients (44%) were eligible for surgery with 91% of R0.
[23]	Nanda, R.H.	2015	29	Retrospective study	In a series of 29 patients treated with FOLFIRINOX, 41.3% of patients underwent surgery with 83% of R0.
[24]	Marthey, L.	2015	77	Multicentre phase II trial	Using RECIST (Response Evaluation Criteria in Solid Tumors) criteria, patients after neoadjuvant FOLFIRINOX had a resection rate of 36% with a 86% of R0.
[12]	Suker, M.	2016	315	Systematic review	Neoadjuvant FOLFIRINOX lead to a 25.9% surgical resection with an R0 resection reported in 60 (74%) patients. The median overall survival was 24.2 months.
[24]	Gemenetzis	2019	415	Retrospective	NAD followed by surgery in LA-PDAC patients improve outcomes in a selected group of patients

**Table 2:** Main characteristics and findings of studies reporting data about NAD in BR-PDAC.

Ref. n°	Author	Year	N° of Patients	Type	Concept Resumed
[26]	Landry, J.	2010	21	Randomized phase II study	Gemcitabine with concomitant RT led to a resection rate of 30% with overall survival of 19.4 months compared to a resection rate of 18% with an OS of 13.4 months when chemotherapy was given alone and followed by RT
[27]	Kim, E.J.	2013	68	Multicentre phase II trial	When Gemcitabine + Oxaliplatin with 30 Gy radiotherapy was given a resection rate of 63% with a R0 resection rate of 84% was found. An OS of 18.2 months was observed
[28]	Katz, M.H.G.	2016	22	Prospective multicenter single-arm trial	Patients treated with modified FOLFIRINOX followed by 5.5 week of RT showed a 68% of resection with an R0 in 14 patients (93%). An OS of 21.7 months was found that represent the highest OS in BR-PDAC.
[29]	Murphy, J.E.	2018	48	Phase 2 clinical trial	Borderline resectable PDAC after 8 cycles of FOLFIRINOX had a resection rate of 66% with an R0 of 97%.
[7]	Versteijne, E.	2020	246	Randomized phase III trial	After gemcitabine based preoperative radiochemotherapy, at the preliminary results, OS in the neoadjuvant group was 17.6 months compared to 13.2 months in the upfront surgery group as well as a higher disease-free survival, lower rates of pathologic lymph nodes.
[30]	Versteijne, E.	2022	246	Randomized phase III trial	5-year OS rate was 20.5% in neoadjuvant chemoradiotherapy group and 6.5% in the upfront surgery one in both resectable and BR-PDAC.

**Table 3:** Main characteristics and findings of studies reporting data about NAD in resectable PDAC.

Ref n°	Author	Year	N° of Patients	Type	Concept Resumed
[7]	Versteijne, E.	2020	246	Randomized phase III trial	At the preliminary results, no differences in OS, DFS, and resection rate were found in resectable PDAC (median OS 14.6 in NAD cohort vs. 15.6 in the upfront surgery one). Resection rate and DFS were also comparable.
[30]	Versteijne, E.	2022	246	Randomized phase III trial	5-year OS rate was 20.5% in the neoadjuvant chemoradiotherapy group and 6.5% in the upfront surgery one in both resectable and BR-PDAC.
[32]	Motoi, F.	2019	360	Multicenter randomized trial	A higher OS after gemcitabine-based neoadjuvant chemotherapy was found. No differences in terms of resection rate, margin status, and morbidity were found.
[33]	Heinrich, S.	2008	28	Nonrandomized prospective study	After preoperative Gemcitabine, resection rate was 89% with 80% of R0, median OS of 26.5 months
[34]	O’Reilly, E.M.	2014	38	Single-arm, nonrandomized phase II trial	Patients treated with neoadjuvant Gemcitabine-Oxaliplatin had an OS of 27.2 months
[35]	Golcher, H.	2015	66	Prospective randomized phase II trial	Neoadjuvant treatment composed by Gemcitabine/cisplatin do not statistically significantly reduced the resection rates but increased the median OS (25 vs. 18.9 months).
[36]	Casadei, R.	2015	38	Single-Center Prospective, Randomized, Controlled Trial	Neoadjuvant chemoradiation was safe but no differences in resection rates and OS were found.

## Data Availability

Not applicable.
